# Analysis of anti-rheumatic activity of *Nyctanthes arbor-tristis* via *in vivo* and pharmacovigilance approaches

**DOI:** 10.3389/fphar.2023.1307799

**Published:** 2023-12-05

**Authors:** Ayushi Sharma, Anjana Goel, Zhijian Lin

**Affiliations:** ^1^ Department of Biotechnology, Institute of Applied Sciences and Humanities, GLA University, Mathura, Uttar Pradesh, India; ^2^ Department of Clinical Chinese Pharmacy, School of Chinese Materia Medica, Beijing University of Chinese Medicine, Beijing, China

**Keywords:** Harsingar, anti-inflammatory, rheumatoid arthritis, cytokines, Freund’s complete adjuvant, indomethacin

## Abstract

**Introduction:** Rheumatoid arthritis (RA) is an immune-mediated disease associated with chronic inflammation of numerous joints. *Nyctanthes arbor-tristis* (NAT) is a traditional remedy for RA, a chronic inflammatory disorder.

**Aim:** The current project aims to demonstrate the role of the NAT extracts in sub-acute toxicity, pharmacovigilance, and anti-rheumatic biomarkers.

**Method:** Hydroethanolic extract (1:1) of plant leaves was prepared by using the reflux method. The safety of the dose was evaluated in Sprague–Dawley rats, and the anti-inflammatory effects of NAT on RA symptoms, including paw volumes, body weight, arthritic index, withdrawal latency, hematology and serological test, radiology, and histopathology, were evaluated in Freund’s complete adjuvant (FCA)-induced arthritis Sprague–Dawley rat models. The inflammatory (TNF-α and COX-2) and anti-inflammatory markers (IL-10) were analyzed in control and experimental groups.

**Result:** The study showed that 500 mg/kg BW NAT leaf extract was found to be least toxic without showing any subacute toxicity symptoms. The pharmacovigilance study highlighted the potential side effects of NAT, such as drowsiness, sedation, and lethargy, at high dosages. Treatment with the plant extract mitigated paw edema, restored the immune organ and body weights, and ameliorated the level of blood parameters such as hemoglobin, red blood cells, platelets, white blood cells, aspartate aminotransferase (AST), alanine transaminase (ALT), C-reactive proteins, and rheumatoid factor. Treatment with the plant extracts also reduced the level of cyclooxygenase 2 and TNF-α and increased the level of IL-10 in the serum of arthritic rats dose-dependently. Radiographic analysis of the ankle joint showed an improvement in the hind legs. Histological examination of the ankle joints revealed that the plant extract treatment decreased pannus formation, inflammation, and synovial hyperplasia in arthritic animals.

**Conclusion:** NAT 500 mg/kg could serve as a promising therapeutic option for the treatment of inflammatory arthritis.

## Graphical Abstract

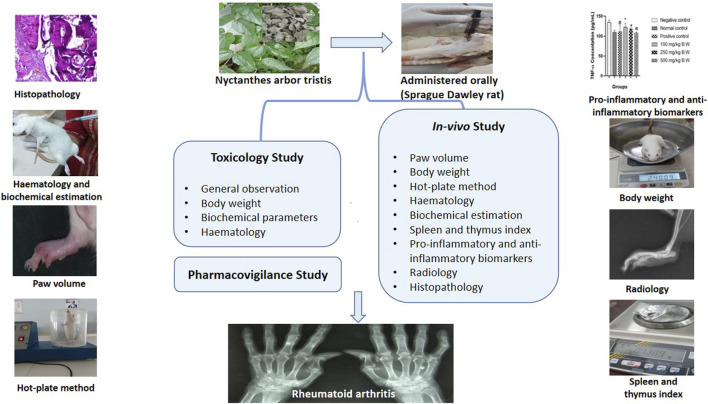

## 1 Introduction

Rheumatoid arthritis (RA) is an immune-mediated disease associated with chronic inflammation of numerous joints. RA symptoms include persistent joint synovial inflammation, pannus development, and subsequent destruction of adjacent bone/cartilage tissue (Marsh et al., 2021). It can rapidly progress to inflammation, affecting various systems with irreversible joint dysfunction, resulting in early mortality, debility, reduced quality of life, and even mortality (Radu and Bungau, 2021). Articular cartilage hyperplasia is a key step in the pathogenesis of RA, which is facilitated by macrophages, T and B lymphocytes, fibroblasts, and pro-inflammatory cytokines, most prominently interleukins (IL-6, IL-1, and TNF-α) (Nygaard and Firestein, 2020). Increased oxidative stress, along with an increase in pro-inflammatory cytokines, is thought to be a significant risk factor for joint deterioration in RA. When inflammatory cells like macrophages and neutrophils stimulate cytokine production, they release reactive oxygen species (ROS) and may damage tissues (Warmink et al., 2023).

Traditional non-steroidal anti-inflammatory medicines (NSAIDs) and disease-modifying anti-rheumatic drugs (DMARDs) have been replaced by innovative biological agents such as TNF monoclonal antibodies, which have improved the treatment strategies for RA patients in recent years (Ben Mrid et al., 2022). Existing medications for rheumatoid arthritis have demonstrated restricted effectiveness in achieving remission in specific patients and are associated with a range of side effects, encompassing systemic organ toxicity affecting the gastrointestinal tract, skin, and kidneys, as well as immunotoxicity, which heightens the risk of infections (Martu et al., 2021). Herbal therapies have been used conveniently for some time with no obvious toxicity or adverse effects, prompting a fresh wave of research into conventional methods currently. Sharma and Goel (2023) suggest that now is the time to look for new and improved natural medicines that can be used for the long-term treatment of RA.

Night jasmine, scientifically known as *Nyctanthes arbor-tristis* Linn and commonly referred to as night jasmine, belongs to the Oleaceae family (Solanki et al., 2021). This plant species is indigenous to South Asia, with its presence documented in regions spanning India (Assam, Arunachal Pradesh, and the area extending southward to the central region of Godavari), Nepal, Bhutan, Java, Sri Lanka, and Sumatra (Pundir et al., 2022). In Ayurveda, *Nyctanthes arbor-tristis* holds significant medicinal potential. It has been identified as a valuable source of bioactive compounds that can serve both as medicines and as intermediates for the development of novel therapeutic agents in modern medicine (Wang et al., 2023). Historically, *Nyctanthes arbor-tristis* has demonstrated a wide range of therapeutic properties, including antimicrobial, antioxidant, antiviral, antidiabetic, antimalarial, antifungal, anti-inflammatory, anticancer, central nervous system depressant, hepatoprotective, and immunostimulant activities. Comprehensive studies, both *in vitro* and *in vivo*, have scientifically validated the presence of these pharmacologically active constituents throughout various parts of the plant (Rawat et al., 2021). Traditionally, the leaves of this plant have been used to prepare decoctions and juices for treating inflammatory disorders such as arthritis and rheumatism. Additionally, the flowers of *Nyctanthes arbor-tristis* have been recognized for their beneficial properties in addressing conditions such as indigestion, flatulence, and stomachic issues; promoting bowel regularity; addressing piles; and managing various skin ailments (Chakraborty and Datta, 2022). In the realm of rheumatic joint pain relief, the powdered stem bark has historically been employed, albeit with limited scientific documentation. The objective of this study was to assess the potential toxicity resulting from the daily administration of hydroethanolic extracts derived from the *Nyctanthes arbor-tristis* leaves for over a 28-day period. Additionally, we aimed to investigate their *in vivo* anti-edematous and anti-arthritic activities as well as their impact on cytokine profiles in a model of Freund's complete adjuvant (FCA)-induced experimental arthritis.

## 2 Materials and methods

### 2.1 Authentication and procurement of the plant

From January to March, leaves were collected from the GLA University campus in India. At the Agharkar Research Institute in Pune, India, the plant specimens were identified and authenticated using the voucher specimen (AUTH 22-48). Fresh leaves were thoroughly washed in distilled water and tap water before being air-dried. When not in use, the dried leaves were coarsely crushed with an electric mixer and stored in sealed plastic bags.

### 2.2 Preparation of hydroethanolic extract

The phytoconstituents were extracted using a hydroethanolic solvent in a 1:1 ratio, employing a reflux apparatus. The resulting extract was then concentrated at 45°C by using a rotary vacuum evaporator from Yamato Scientific Co., Japan. In preparation for the *in vivo* study, the concentrated extract was subsequently stored at −20°C.

### 2.3 Experimental animal

The study protocol was approved by the Institutional Animal Ethical Committee (IAEC) of the Dept. of Biotechnology and Institute of Pharmaceutical Research, GLA University, Mathura, India, in accordance with the regulations of CPCSEA (1260/PO/AC/09/CPCSE). Female Sprague–Dawley (SD) rats were obtained from the “National Institute of Biologicals,” Noida, India. The animals were housed in a typical animal housing facility with a temperature of 24 ± 1°C, relative humidity of (45%–50%), and a light/dark cycle of 12 h. The animals were fed a conventional pellet chow diet and were given unlimited access to water. The animals were allowed to acclimate for at least 10 days before the experiments.

### 2.4 Selection of doses and sub-acute toxicity studies

The dose was chosen based on prior toxicity tests, which showed that a dose of 2 g/kg BW did not cause mortality in rats (Hazarika et al., 2022). As a result, the maximal dose in this investigation was set at 2 g/kg BW. The oral toxicity investigation was conducted based on OECD Guideline 407 (Balkrishna et al., 2023). The animals were divided into six groups of six each (females). Group 1 was treated as the vehicle control. Extract doses of 100, 250, 500, 1,000, and 2,000 mg/kg/BW were administered to groups 2, 3, 4, 5, and 6 and the vehicles, respectively, for 28 days. All the extracts were given via oral gavage.

#### 2.4.1 General observation and mortality

Death, any sign of illness, and response to therapy were recorded twice daily, along with other general observations like changes in the skin, drowsiness, sedation, eye color, general physique, coma, and death. The procedures were conducted following the guidelines for the recognition, evaluation, and use of clinical signs as the human endpoint for the safety evaluation of experiments involving animals.

#### 2.4.2 Body weight

During the experiment, the body weight was recorded at 0 days and after 7-day intervals up to 28 days by using a digital weighing balance.

#### 2.4.3 Hematological and biochemical analysis

All the animals were made to fast overnight before drawing the blood for analysis of the hematology and biochemical parameters. The animals were provided free access to water. The rats were anesthetized before drawing blood samples directly from the heart, and the samples were stored in EDTA-coated and plain vials. Hemoglobin, white blood cell count, red blood cell count, platelets, protein, albumin, gamma globulin, urea, uric acid, creatinine, AST, ALT, bilirubin, and cholesterol of the control and plant-treated groups were estimated using an automatic hematology analyzer (hematology auto analyzer MEK-6420P) and clinical chemistry analyzer (semi-automated biochemistry analyzer Erba Chem 5X).

### 2.5 Pharmacovigilance study

In this research, we conducted a pharmacovigilance study, including the detection, assessment, understanding, and prevention of the potential risk of NAT, through literature reviewing*, in vivo* experiments, and surveillance database mining.

#### 2.5.1 Detection

This involves identifying potential adverse drug reactions (ADRs) associated with the use of *Nyctanthes arbor-tristis*. This can be done through spontaneous reporting, clinical trials, systematic reviews, and observational studies. There is limited scientific evidence available on the potential ADRs associated with *Nyctanthes arbor-tristis*, as it is a traditional herbal medicine and has not been extensively studied in clinical trials. From the Chinese Field Herbarium official website (http://www.cfh.ac.cn/), the term *Nyctanthes arbor-tristis* was entered, and the relevant species information card (http://www.cfh.ac.cn/34781.sp) was used to find the nickname of *Nyctanthes arbor-tristis*. With regard to the detection of potential safety issues, this study systematically searched the China National Medical Product Administration website, the FDA Adverse Event Reporting System, the Canadian Institutes of Health Research, and the Medicinal plant database, Botanical Survey of India, India (https://bsi.gov.in/page/en/medicinal-plant-database) and comprehensively identified adverse reactions associated with *Nyctanthes arbor-tristis*. In addition, we also searched published articles from PubMed, China National Knowledge Infrastructure Database, and Google Scholar to detect the potential risk of *Nyctanthes arbor-tristis*.

#### 2.5.2 Assessment

After identifying the ADRs of *Nyctanthes arbor-tristis*, further research should be conducted to assess the severity and frequency of these reactions. Since *Nyctanthes arbor-tristis* is a traditional herb commonly used mostly in India and China, and there is no patent drug made from just *Nyctanthes arbor-tristi*s, we searched the database of clinical trials in China (http://www.chictr.org.cn), NIH U.S. National Library of Medicine (https://clinicaltrials.gov), and India to conduct the risk assessment.

#### 2.5.3 Understanding

Based on the literature search, there are some potential ADRs related to the risks of *Nyctanthes arbor-tristis*, and this research conducted a safety experiment to find out the mechanism of the risk of *Nyctanthes arbor-tristis*. This study promoted the understanding of the potential risk of *Nyctanthes arbor-tristis*. Further research can be conducted to understand the mechanisms behind these reactions and the risk factors for developing them.

#### 2.5.4 Prevention

Based on the results of the risk assessment, appropriate risk minimization strategies are developed and implemented. Based on the pharmacovigilance study, we should pay attention to the dosage and duration of *Nyctanthes arbor-tristis* in medical use. If there is any adverse reaction monitored, it will be necessary to discontinue the use of *Nyctanthes arbor-tristis* and treat the ADR symptoms.

### 2.6 Anti-rheumatic activity

#### 2.6.1 Induction of RA by FCA

Subcutaneous injection of approximately 0.1 mL of FCA was administered to the subplantar region of the left hind paw on both the first and seventh days to all the animals in every group, except for those in the vehicle control group (Singh et al., 2021).

For the study of FCA-induced arthritis, the rats were split into six groups with six rats in each group.

Group I: vehicle control: normal saline was given as an oral 1% w/v suspension.
Group II: arthritic control: normal saline was given as an oral 1% w/v suspension.
Group III: arthritic animals were given 10 mg/kg of body weight of indomethacin, a commonly used anti-inflammatory medication.
Group IV: rheumatic arthritic animals were treated orally with a hydroethanolic fraction of NAT at a dose of 100 mg/kg body weight.
Group V: rheumatic arthritic animals were treated orally with a hydroethanolic fraction of NAT at a dose of 250 mg/kg body weight.
Group VI: rheumatic arthritic animals were treated orally with a hydroethanolic fraction of NAT at a dose of 500 mg/Kg body weight.

At the conclusion of the experiment, the rats were anesthetized using 7% chloral hydrate (400 mg/kg, administered intraperitoneally; supplied by Sinopharm Chemical Reagent Co., Ltd.). It was verified that the rats exhibited no indications of peritonitis, pain, or discomfort as a result of anesthesia. Following anesthesia, blood was drawn from the live anesthetized rats from the abdominal aorta. After obtaining 5 mL of blood, the rats were subsequently euthanized through cervical dislocation. Furthermore, the ankle joints, synovial tissue, and spleen were collected from each animal.

#### 2.6.2 Evaluation of paw volume

A "plethysmometer (Ugo, Basile, Italy)" was used to measure the left paw volume up to the lateral malleolus before FCA injection on the first day and at 7-day intervals thereafter until the 28th day (Shakeel et al., 2021).

#### 2.6.3 Determination of body weight

During the experiment, the body weight was measured by using a digital scale (Sartorius 1413, MP 8/8-1, Bohemia, NY, United States) before the first FCA injection on the first day and then at different times until the 28th day (Singh et al., 2021).

#### 2.6.4 Analgesic assessment using the hot plate test

Analgesic activity was evaluated using a modified version of Eddy’s hot plate method (Gurung et al., 2020). The temperature of the plate used to house the rats was maintained at 55 ± 1°C. There was a log detailing how long the rats took for paw licking and how long they took for jumping. Each rat’s reaction time, measured in seconds, was recorded as it took flight from the plate. The nociceptive response was assessed at 15-min intervals for 90 min before and after the administration of the vehicle; NAT hydroethanolic extracts at 100, 250, and 500 mg/kg and indomethacin at 10 mg/kg.

#### 2.6.5 Radiological analysis of ankle joints

On day 28, after injecting FCA into the animal’s hind paws, they were anesthetized using ketamine anesthesia for X-rays analyzed by (GE DX-300). At 40 kV peak and 12 Ms, radiographs of the hind paw were taken. The radiographic alterations were deduced from the X-ray image (Alamgeer et al., 2017).

#### 2.6.6 Determination of spleen and thymus weight

Rats were slaughtered using ketamine anesthesia after the experiments. The weights of all the thymus and spleens were measured.

#### 2.6.7 Parameter of biochemical estimation

The blood was taken on the 28th day of the experiment. Aspartate aminotransferase (AST), alanine transaminase (ALT), C-reactive protein (CRP) level, and rheumatoid factor (RF) were estimated in the serum (Vijeesh et al., 2022).

#### 2.6.8 Estimation of inflammatory biomarkers (TNF-α, COX-2, and IL-10)

Pro-inflammatory and anti-inflammatory biomarkers, including TNF-α (DY510-05), COX-2 (ELK7718), and IL-10 (DY522-05), were analyzed using the R&D Systems DuoSet Development Kit with pre-made ELISA reagent kits as per the instruction of the manual by ELISA (i-mark microplate absorbance reader Bio-Rad (Felipe et al., 2020).

#### 2.6.9 Histopathological assessment of joints

For histological analysis, the paws were removed from treated and control rats and their ankle joints were transacted between the medial and lateral malleoli. The rats’ feet were then placed in 10% formalin (Uttra et al., 2019).

### 2.7 Data and statistical analysis

The data are presented in the form of mean ± SEM for a sample size of six animals. We conducted statistical analysis on the experimental results using one-way ANOVA followed by the Dunnett test, employing GraphPad InStat software. A significance level of *p* < 0.05 was employed to determine statistical significance.

## 3 Result

### 3.1 Sub-acute toxicity study

The sub-acute toxic study of the tested plant extract was determined as per OECD guideline 407.

#### 3.1.1 General observation and mortality

In the experiment groups and the control group, no treatment-related deaths were reported. Throughout the 28-day study period, neither physical nor behavioral changes were seen in the groups, but drowsiness, sedation, and lethargy were observed in 1,000 and 2,000 mg/kg BW animals, as shown in Table 1.

**TABLE 1:** General appearance and behavioral observations of the sub-acute toxicity study for the control and treated groups.

Groups	Change in skin	Drowsiness	Sedation	Eye color	General physique	Coma	Death
Vehicle control	No effect	Not present	Not observed	No effect	Normal	Not present	Alive
NAT (100 mg/kg)	No effect	Not present	Not observed	No effect	Normal	Not present	Alive
NAT (250 mg/kg)	No effect	Not present	Not observed	No effect	Normal	Not present	Alive
NAT (500 mg/kg)	No effect	Not present	Not observed	No effect	Normal	Not present	Alive
NAT (1,000 mg/kg)	No effect	Present	Observed	No effect	Lethargy	Not present	Alive
NAT (2,000 mg/kg)	No effect	Present	Observed	No effect	Lethargy	Not present	Alive

#### 3.1.2 Change in body weight

There was no statistically significant difference found in average body weight between the vehicle control and the NAT group at doses of 100, 250, 500, 1,000, and 2,000 mg/kg, but in the 1,000 and 2,000 mg/kg BW group animals, decrease in body weight was observed compared to other groups. The effect of NAT extract on body weight is shown in Table 2 (*p* > 0.05).

**TABLE 2:** Effect of different concentrations of NAT extracts on body weight.

Groups	Body weight on different days (g)					
	Day 0	Day 7	Day 14	Day 21	Day 28	Change in % (Days 0–28)
Vehicle control	212.3 ± 23.6	210.5 ± 21.3	216.5 ± 22.7	218.6 ± 23.2	216.1 ± 21.9	1.78
NAT (100 mg/kg)	234.66 ± 22.6	239 ± 24.3	245.3 ± 25.5	246.83 ± 22.9	247.3 ± 24.9	5.38
NAT (250 mg/kg)	217.33 ± 7.2	218.4 ± 7.3	234.5 ± 12.7	235.7 ± 11.3	234.4 ± 10.9	7.85
NAT (500 mg/kg)	212.5 ± 14.9	226.8 ± 16.3	243.813.8	247.4 ± 14.7	248 ± 11	16.70
NAT (1,000 mg/kg)	215 ± 40.8	223.8 ± 39.9	213 ± 43.6	198.5 ± 39.9	195.6 ± 37.6	−9.02
NAT (2,000 mg/kg)	240.33 ± 22.23	236 ± 17.2	220.33 ± 39.2	212.5 ± 39.9	208.4 ± 37.5	−13.28

#### 3.1.3 Effect of the plant extract on hematological and biochemical parameters

The results of the various hematological and biochemical parameters tests on the experimental and vehicle groups are summarized in (Tables 3, 4, and 5). Oral administration of NAT at doses of 100, 250, 500, 1,000, and 2,000 mg/kg did not cause statistically significant changes in hematological and biochemical parameters, such as protein, albumin, gamma-globulin, urea, sodium, creatinine, uric acid, SGOT (AST), SGPT (ALT), bilirubin, and total cholesterol levels, when compared to the control group, but increased levels of SGOT, SGPT, bilirubin, and total cholesterol showed toxicity were observed when compared to the vehicle control.

**TABLE 3:** Renal profile of the control group and rats treated with NAT leaf extract measured during the sub-acute toxicity study.

Parameters	Vehicle control	NAT (100 mg/kg)	NAT (250 mg/kg)	NAT (500 mg/kg)	NAT (1,000 mg/kg)	NAT (2,000 mg/kg)
Urea (mg/dL)	30.33 ± 4.72	32.2 ± 5.40	30.7 ± 5.70	34.74 ± 7.31	35.44 ± 6.10	35.17 ± 2.50
Uric acid (mg/dL)	3.73 ± 0.75	3.55 ± 0.71	3.95 ± 0.62	3.87 ± 0.44	3.89 ± 0.25	4.04 ± 0.36
Creatinine (mg/dL)	0.71 ± 0.14	0.68 ± 0.12	0.72 ± 0.10	0.62 ± 0.14	0.73 ± 0.13	0.85 ± 0.06

**TABLE 4:** Liver profile of the control group and rats treated with NAT leaf extract measured during the sub-acute toxicity study.

Parameters	Vehicle control	NAT (100 mg/kg)	NAT (250 mg/kg)	NAT (500 mg/kg)	NAT (1,000 mg/kg)	NAT (2,000 mg/kg)
SGOT (AST) (IU/L)	162 ± 37.24	168.5 ± 41.41	163 ± 20.92	167.5 ± 39.68	178.7 ± 66.70	214.5 ± 42.65
SGPT (ALT) (IU/L)	134.66 ± 40.5	142.04 ± 38.83	143.6 ± 16.5	143.75 ± 42.30	165.4 ± 47.17	181 ± 22.32
Bilirubin (g/dL)	0.40 ± 0.09	0.45 ± 0.16	0.58 ± 0.15	0.58 ± 0.1	0.62 ± 0.05	0.64 ± 0.10
Total cholesterol (mg/dL)	96.43 ± 9.22	93.75 ± 16.98	98.74 ± 8.38	98.9 ± 12.72	108.24 ± 52.6	116.52 ± 48.5

**TABLE 5:** Hematological parameters of the control group and rats treated with NAT leaf extract measured during the sub-acute toxicity study.

Parameters	Vehicle control	NAT (100 mg/kg)	NAT (250 mg/kg)	NAT (500 mg/kg)	NAT (1,000 mg/kg)	NAT (2,000 mg/kg)
`Q Protein (g/dL)	6.67 ± 0.64	6.28 ± 0.42	6.42 ± 0.36	6.39 ± 0.38	6.59 ± 0.88	6.77 ± 0.53
Albumin (g/dL)	3.9 ± 0.57	3.52 ± 0.48	3.66 ± 0.38	3.80 ± 0.09	3.84 ± 0.76	3.33 ± 0.32
γ-Globulin (g/dL)	3.34 ± 0.37	2.62 ± 0.72	2.62 ± 0.46	2.49 ± 0.52	3.07 ± 0.24	2.93 ± 0.58
Hemoglobin (g/L)	11.96 ± 0.28	12.93 ± 0.4	13 ± 0.5	14.4 ± 0.2	12.35 ± 0.38	11.5 ± 0.7
WBC (10^9^/L)	9733.33 + 1150	9830 + 2600	9600 + 731.81	9533.33 + 500	9800 + 1699	9950 + 1681.93
Total RBC (10^12^/L)	8.62 ± 0.51	8.74 ± 1.52	9.05 ± 0.71	10.17 ± 0.93	10.21 ± 1.47	9.14 ± 1.03
Platelets (10^3^/𝜇L)	708.40 ± 117.74	881.70 ± 56.09	764.50 ± 272.37	835.00 ± 290.60	718.67 ± 111.74	697.88 ± 69.91

### 3.2 Pharmacovigilance finding and measures

*Nyctanthes arbor-tristis* is a traditional herbal medicine commonly used in China, India, and other Asian countries. It is very important to conduct pharmacovigilance research for *Nyctanthes arbor-tristis.* Based upon the theory, science, and activities of pharmacovigilance, conducting pharmacovigilance research for *Nyctanthes arbor-tristis* involves several steps given as follows

#### 3.2.1 Detection

On the PubMed website (https://pubmed.ncbi.nlm.nih.gov/), the terms *Nyctanthes arbor-tristis* Linn, Bruschia macrocarpa Bertol, *Nyctanthes* dentata Blume, *Nyctanthes tristis* Salisb, *Parilium arbor-tristis* (L.) Gaertn, *Scabrita scabra* L, and *Scabrita triflora* L were entered and relevant literature on adverse reactions was searched for one by one. Some possible ADRs that have been reported in traditional medicine practices include gastrointestinal complaints such as stomach pain, gastric ulcer, nausea, vomiting, and diarrhea, which have been reported in some cases (Saxena et al., 1987; Godse et al., 2016); allergic reactions such as skin rash, itching, and hives have been reported in some individuals; central nervous system effects such as drowsiness, headache, and dizziness have been reported in some cases; some cases also report liver and kidney damage (Das et al., 2008). It is important to note that these ADRs are based on traditional medical practices and may not have been rigorously studied in clinical trials. In addition, there are no adverse drug reactions in the ADR monitoring system in China, the United States, Canada, and India. Therefore, it is essential to use caution when using *Nyctanthes arbor-tristis* and to consult with a healthcare professional before taking this herbal medicine.

#### 3.2.2 Assessment

No clinical trial registration was found. *Nyctanthes arbor-tristis* is not a patent medication authorized by the China National Medical Products Administration or by the Indian government. There were no post-marketing ADR monitoring data. In this case, it is difficult to conduct the benefit–risk assessment. More data and clinical evidence should be collected for future assessment to balance the benefits and risks of *Nyctanthes arbor-tristis*.

#### 3.2.3 Understanding

In this study, we carried out *in vivo* studies, as well as pharmacokinetic and pharmacodynamic analyses, and we identified the mechanism. In this study, we observed that NAT can cause drowsiness, sedation, and lethargy at the high dosages of 1,000 mg/kg and 2,000 mg/kg. Some other studies indicated that the inhibition of AchE activity is reduced in malathion-treated serum and brain tissue samples of mice treated with NAT extract. The hot infusion of NAT flowers has shown sedative potential in mice. The ethanolic extract of NAT flowers, seeds, and leaves has shown central nervous system depression activity. The potential bioactive compounds might include arbortristoside-A, arbortristoside-B, astragalin, nicotiflorin, and quercetin (Sharma et al., 2021).

#### 3.2.4 Prevention

We have identified certain risks of *Nyctanthes arbor-tristis* with potential ADRs. Hence, we should develop some strategies and measures to prevent or minimize the risks associated with *Nyctanthes arbor-tristis*. We should pay attention to the dosage and duration of the medical use of *Nyctanthes arbor-tristis*. It will be necessary to discontinue the use of *Nyctanthes arbor-tristis* and treat the ADR symptoms when ADRs are monitored. Further research should be conducted for implementing risk mitigation strategies, such as toxic study changes, mechanism and potential risk component identification, patient education or training of healthcare professionals about the safe and rational use of this herbal medication, and even post-marketing surveillance after marketing authorization.

### 3.3 Anti-arthritic activity

#### 3.3.1 FCA-induced arthritis

The subplantar injection of FCA in the left hind paw of the rats resulted in a progressive increase in the volume of the ipsilateral (injected) paw as well as the contralateral (non-injected) paw.

#### 3.3.2 Effect of the extract on paw volume

FCA was administered on the first and third day, which resulted in a progressive increase in paw volume. The treatment with standards (indomethacin) and plant concentration (NAT) started from day 0 and continued to day 28. As presented in Table 6, it can be seen that treatment with standards as well as the plant extract caused significant abatement of paw volume, which was noticed from day 14 to day 28. NAT (500 mg/kg) demonstrated a high level of anti-arthritic effects (63.63%) compared to that of indomethacin (56.92%). However, the anti-arthritic effects of NAT (250 mg/kg) (48.81%) were found to be significantly lower than those of indomethacin and NAT (500 mg/kg). Hence, NAT (500 mg/kg) could be used as an alternative to indomethacin in the treatment of RA.

**TABLE 6:** Effect of the plant extract on Freund’s complete adjuvant (FCA)-induced paw volume of rats.

Groups	Paw volume on different days (mL)					
	Day 0	Day 7	Day 14	Day 21	Day 28	Change in % (Day 0–28)
Arthritic control	0.46 ± 0.08	1.36 ± 0.19	1.31 ± 0.07	1.19 ± 0.11	1.0 ± 0.16	117.39
Vehicle control	0.46 ± 0.08	0.47 ± 0.07	0.49 ± 0.09	0.50 ± 0.10	0.52 ± 0.12^#^	13.04
NAT (100 mg/kg)	0.48 ± 0.09	1.39 ± 0.15	1.30 ± 0.17	0.95 ± 0.18	0.94 ± 0.29	95.83
NAT (250 mg/kg)	0.43 ± 0.08	1.27 ± 0.20	1.13 ± 0.19	0.87 ± 0.14*	0.65 ± 0.10*	51.16
NAT (500 mg/kg)	0.45 ± 0.05	1.32 ± 0.24	0.95 ± 0.15*	0.71 ± 0.12^#^	0.48 ± 0.08^#^	6.66
Positive control	0.43 ± 0.05	1.30 ± 0.15	1.05 ± 0.14*	0.77 ± 0.17*	0.56 ± 0.14^#^	30.23

Values are expressed as mean ± SEM for six animals. Symbols represent statistical significance: ∗ = < 0.05, # = < 0.01, @ = < 0.001 when compared to arthritic control. Bonferroni post-test after a two-way analysis of variance.

#### 3.3.3 Body weight

All animals injected with FCA showed a reduction in body weight, which might be due to the decreased absorption of nutrients through the intestine (Taylor et al., 2009). However, the treatment with standards (indomethacin) and the plant extract showed an increase in the body weight from the 14th day onward. The NAT (500 mg/kg) and NAT (250 mg/kg) were found to restore the body weight and also increase it (5.25% and 2.02%) in a progressive manner, such as in the case of indomethacin (2.93%), as shown in Table 7. Overall, both NAT (500 mg/kg) and NAT (250 mg/kg) were found to have a good impact on the body weight of the rats.

**TABLE 7:** Effect of the plant extract on body weight.

Groups	Body weight on different days (g)					
	Day 0	Day 7	Day 14	Day 21	Day 28	Change in % (Day 0–28)
Arthritic control	252.8 ± 9.2	241.5 ± 5.6	232.7 ± 6.1	229.5 ± 7.6	226.5 ± 9.3	−10.40
Vehicle control	255 ± 6.1	258.2 ± 5.9	262.4 ± 6.1	264.8 ± 5.6	266.8 ± 5.8	4.62
NAT (100 mg/kg)	253.1 ± 22.4	247.2 ± 23.1	246 ± 23.2	244.7 ± 23	245.2 ± 23.4	−3.12
NAT (250 mg/kg)	252.4 ± 5.9	248.7 ± 5.7	249.4 ± 4.9^#^	251.8 ± 5.4^#^	257.5 ± 7^#^	2.02
NAT (500 mg/kg)	251.4 ± 16.1	256.2 ± 15.7	259 ± 15.5^#^	261.8 ± 15.5^#^	264.6 ± 15.6^#^	5.25
Positive control	255.2 ± 10.8	251.6 ± 10.4	252.5 ± 11.1^#^	256.8 ± 11.1^#^	262.7 ± 8.8^#^	2.93

Values are expressed as mean ± SEM for six animals. Symbols represent statistical significance: ∗ = < 0.05, # = < 0.01, @ = < 0.001 when compared to arthritic control. Bonferroni post-test after a two-way analysis of variance.

#### 3.3.4 Hot plate method

The analgesic effect can be assessed in the hot plate test. The effect of NAT at 100–500 mg/kg BW on hot-plate response latency is shown in Table 8. The hot-plate response latency in animals treated with 100, 250, and 500 mg/kg BW NAT was significantly different from that of the negative control. NAT (100 mg/kg) shows significant result from 60 to 90 min (0 < 0.05), and NAT (250 mg/kg) and NAT (500 mg/kg) show significant results from 30 to 180 min (*p* < 0.001). A typical analgesic effect was seen in the NAT group rats.

**TABLE 8:** Effects of *Nyctanthes arbor-tristis* and indomethacin on pain induced by analgesic assessment using the hot plate test.

Groups	Time of reaction (Sec)						
	0 min	30 min	60 min	90 min	120 min	150 min	180 min
Arthritic control	3.83 ± 0.35	4.02 ± 0.30	3.80 ± 0.20	4.07 ± 0.20	3.75 ± 0.30	3.73 ± 0.25	3.53 ± 0.24
Vehicle control	4.00 ± 0.23	4.22 ± 0.60	4.08 ± 0.29	4.15 ± 0.21	4.25 ± 0.41	4.17 ± 0.30	4.17 ± 0.57
NAT (100 mg/kg)	3.88 ± 0.42	4.22 ± 0.53	4.45 ± 0.50*	4.65 ± 0.49*	4.33 ± 0.49*	4.20 ± 0.50	3.98 ± 0.40
NAT (250 mg/kg)	4.10 ± 0.28	5.24 ± 0.70^@^	6.03 ± 0.42^@^	7.35 ± 0.29^@^	6.32 ± 0.33^@^	6.00 ± 0.35^@^	5.28 ± 0.32^@^
NAT (500 mg/kg)	4.28 ± 0.22	5.02 ± 0.29^@^	6.47 ± 0.23^@^	7.68 ± 0.32^@^	6.80 ± 0.22^@^	6.43 ± 0.23^@^	5.78 ± 0.40^@^
Positive control	4.60 ± 0.43^#^	5.05 ± 0.30^@^	5.78 ± 0.56^@^	6.25 ± 0.37^@^	6.85 ± 0.45^@^	6.43 ± 0.50^@^	5.78 ± 0.37^@^

Values are expressed as mean ± SEM for six animals. Symbols represent statistical significance: ∗ = < 0.05, # = < 0.01, @ = < 0.001 when compared to arthritic control. Bonferroni post-test after a two-way analysis of variance.

#### 3.3.5 Radiological analysis of ankle joints

**FIGURE 1:**
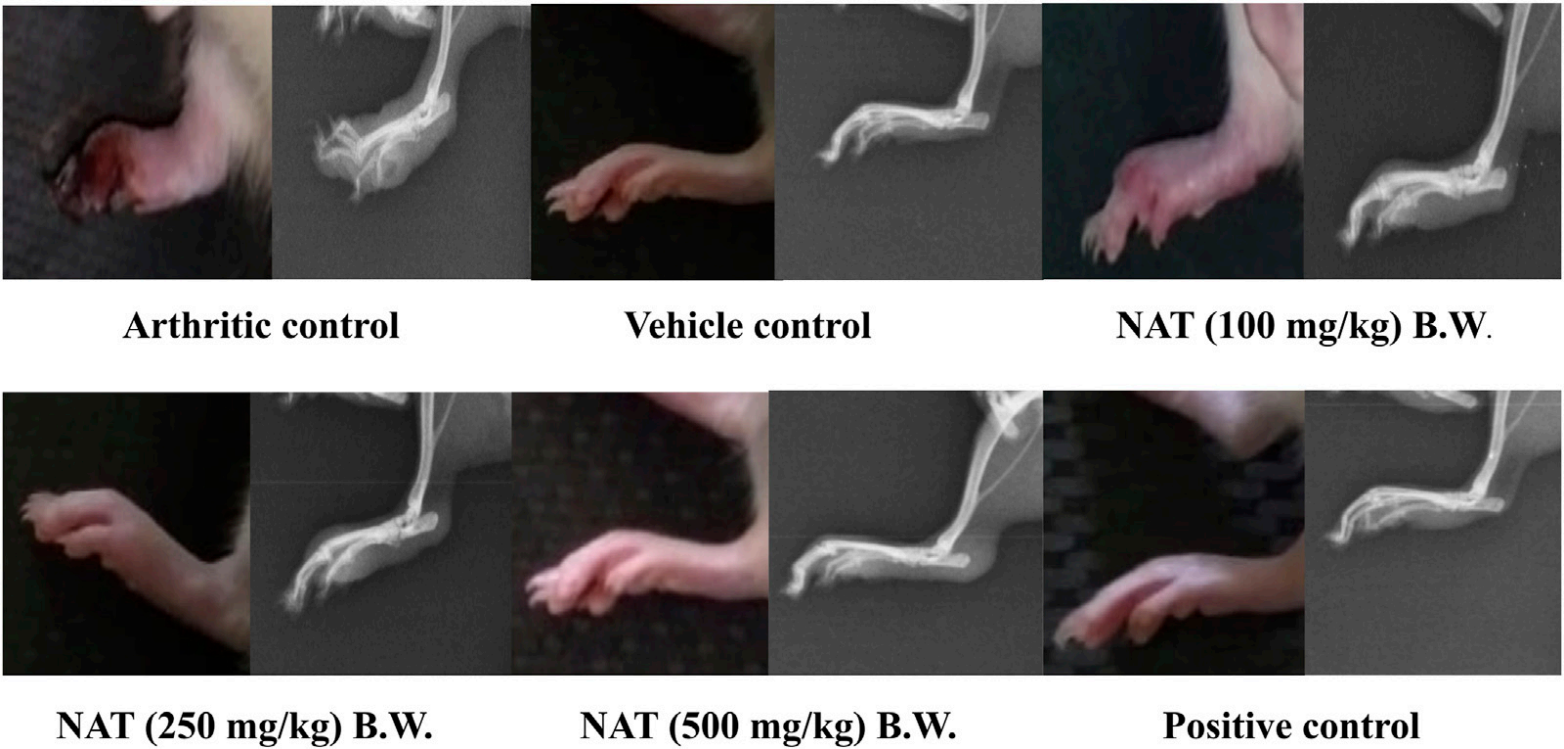
Radiographic analysis of left hind limbs of FCA-induced arthritic rats treated with *Nyctanthes arbor-tristis* extract.

Figure 1 shows the radiographs of the joints in the rats with and without treatment for FCA-induced arthritis. In addition to normal joint spaces and connective tissue, the joints of vehicle control rats did not exhibit focal cartilage bone erosion or joint tissue swelling. All of the treatments had a positive impact on the radiographic changes in the joints of the adjuvant-injected control rats, including extensive phalangeal bone erosion; periarticular bone resorption; no discernible joint spaces; extensive joint deformity; diffuse soft tissue swelling; and thickened, significantly enlarged, and dense connective tissue. The prophylactic administration of NAT at doses of 250 mg/kg and 500 mg/kg significantly reduced the radiographic changes in the arthritic control group. Additionally, NAT (250 mg/kg) treatment moderately reduced bone erosion and resorption as well as noticeable joint deformity. NAT administration (100 mg/kg) had only marginally protective effects. Additionally, indomethacin-treated rats’ radiographs showed a mild-to-moderate level of protection against the radiographic changes seen in the adjuvant control group.

#### 3.3.6 Measurement of spleen and thymus weights

**FIGURE 2:**
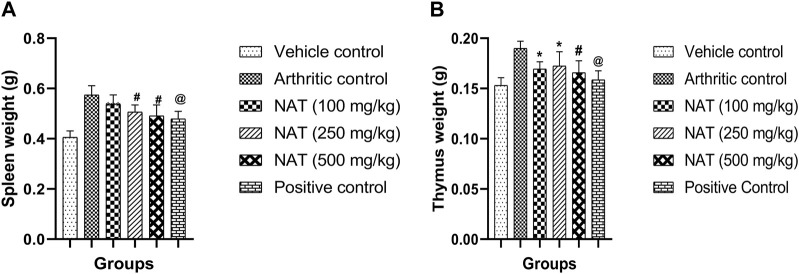
Effect of *Nyctanthes arbor-tristis* extract on spleen **(A)** and thymus **(B)** weights in FCA-induced arthritic rats. Values are expressed as mean ± SEM for six animals. ∗ = < 0.05, # = < 0.01, @ = < 0.001 when compared to arthritic control rats. We performed a one-way analysis of variance followed by the Dunnett test.

Immunological functions are related to the thymus and spleen indexes. On the 28th day, the rats were sacrificed and the thymus index and spleen index were determined. As presented in Figure 2, the weights of the spleen and thymus for the NAT (500 mg/kg) group animals were found to be significantly lower than those of the arthritic control group (*p* < 0.01). NAT (250 mg/kg) and NAT (100 mg/kg) groups also showed increase in thymus weight (0.17 ± 0.014 g and 0.16 ± 0.007 g) but slight decrease in spleen weight (0.50 ± 0.028 g and 0.53 ± 0.036 g). Based on these observations, NAT (500 mg/kg) was found to be much better than NAT (250 mg/kg) in reducing the thymus and spleen weights.

#### 3.3.7 Serum lysosomal enzymes in FCA-induced arthritis

**FIGURE 3:**
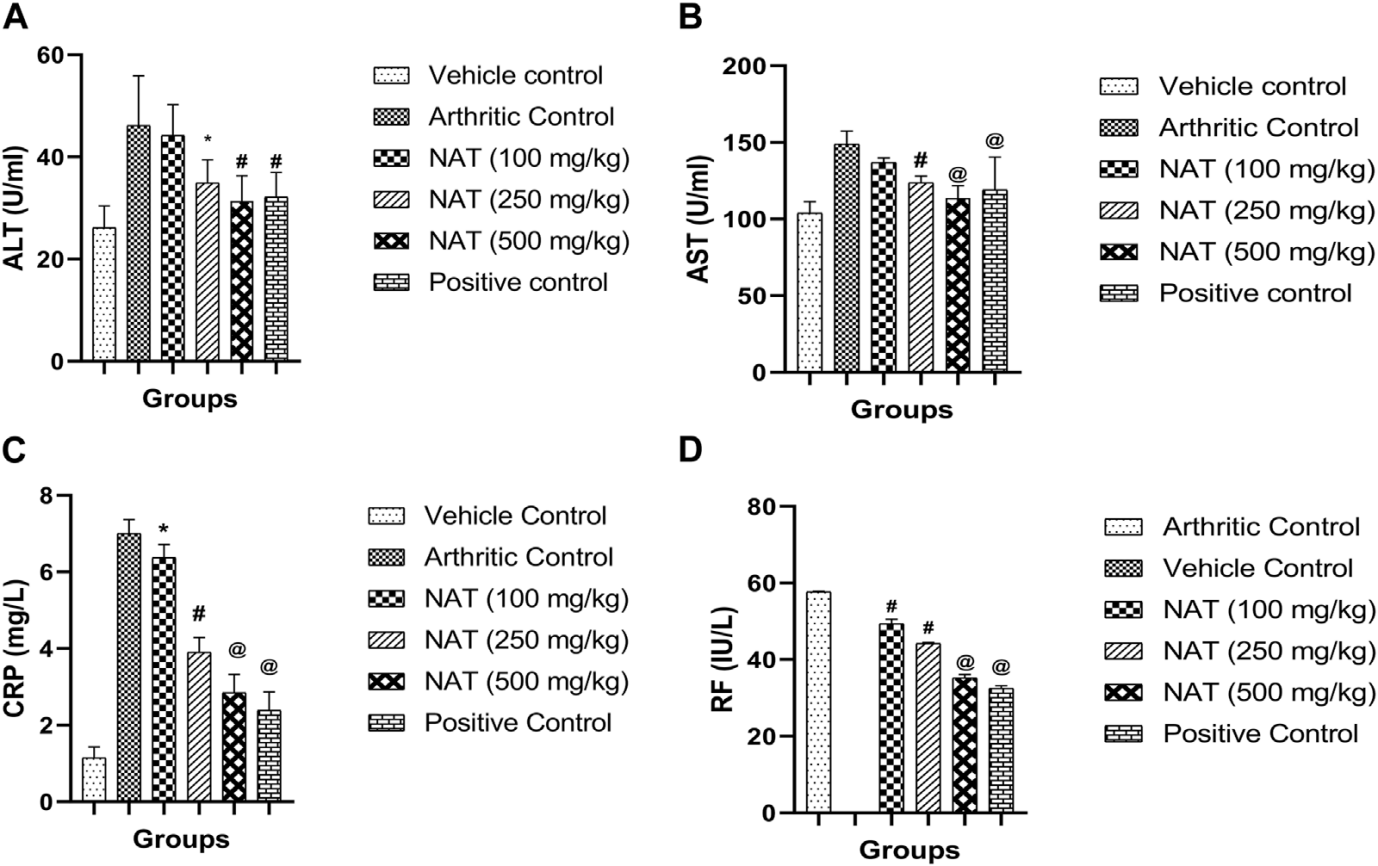
Effect of *Nyctanthes arbor-tristis* extract on serum lysosomal enzymes: ALT **(A)**, AST **(B)**, CRP **(C)** and RF **(D)** in FCA-induced arthritic rats. Values are expressed as mean ± SEM for six animals. ∗ = < 0.05, # = < 0.01, @ = < 0.001 when compared to arthritic control rats. We performed a one-way analysis of variance followed by the Dunnett test.

During the inflammatory response, enzymes like alanine aminotransferase (AST) and alanine transaminase (ALT) play crucial roles in the production of chemical mediators like bradykinins (Cheng et al., 2015). In addition, serum alanine aminotransferase (AST) and alanine aminotransferase (ALT) are specific biomarkers helpful in assessing liver damage (Agrawal and Pal, 2013). Therefore, AST and ALT concentrations were assessed. The FCA treatment increased enzyme levels in all animal groups, and serum CRP and RF are the markers of inflammation and the creation of antibodies against the injected FCA. In the FCA control group animals, high levels of CRP (7.0 mg/L) and RF (57.68 IU/L) were found. However, as shown in Figure 3, NAT (500 mg/kg), NAT (250 mg/kg), NAT (100 mg/kg), and indomethacin (10 mg/kg) markedly decreased the levels of AST, ALT, CRP, and RF. NAT (500 mg/kg) treatment was more effective than that of NAT (250 mg/kg) in lowering the serum levels of AST, ALT, CRP, and RF.

#### 3.3.8 Hematological parameters in FCA-induced arthritis

**FIGURE 4:**
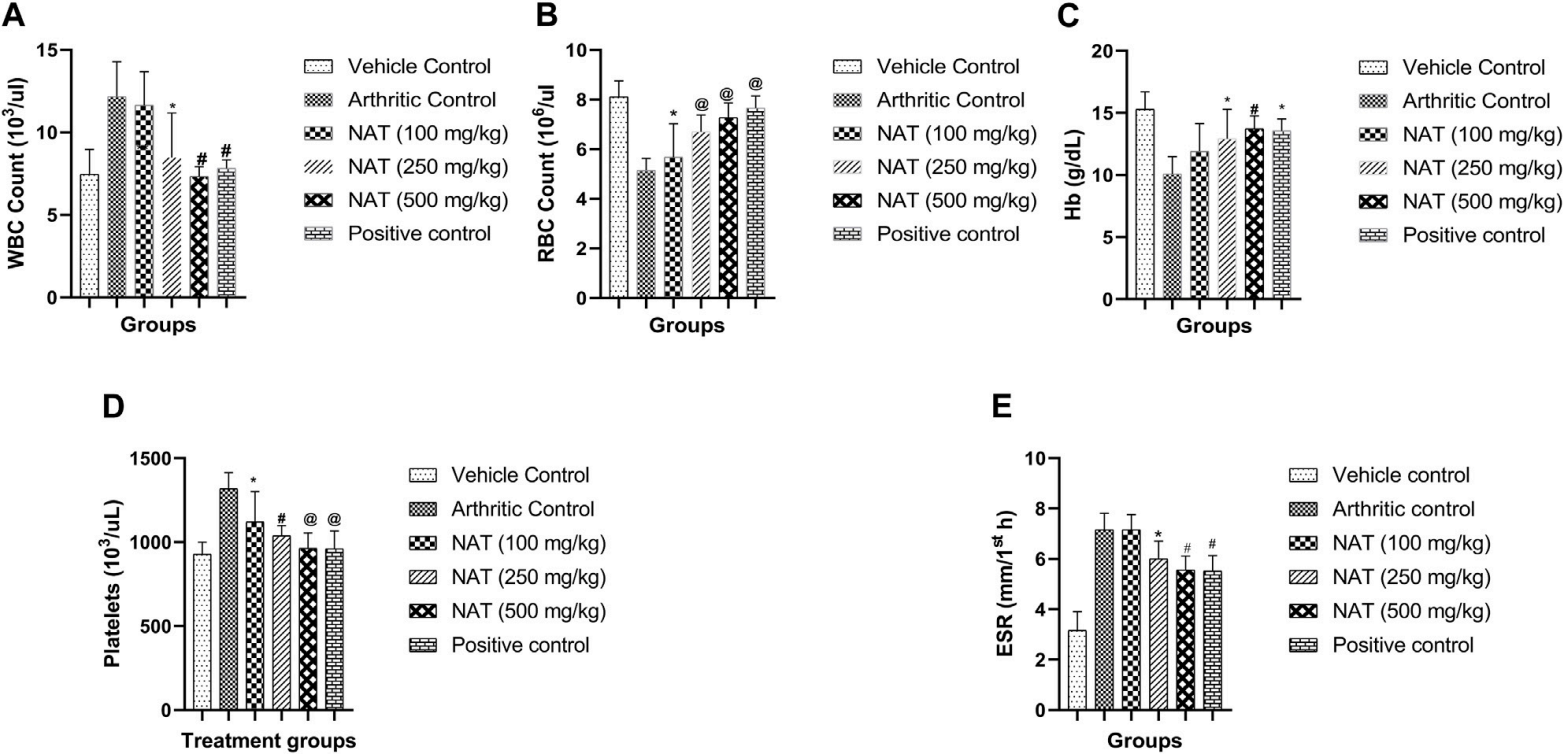
Effect of *Nyctanthes arbor-tristis* extract on Hematological parameters: WBC **(A)**, RBC **(B)**, Hb **(C)**, Platelets **(D)** and ESR **(E)** in FCA-induced arthritic rats. Values are expressed as mean ± SEM for six animals. ∗ = < 0.05, # = < 0.01, @ = < 0.001 when compared to arthritic control rats. We performed a one-way analysis of variance followed by the Dunnett test.

When comparing arthritic control rats to other groups, NAT (500 mg/kg) showed a significant decrease (*p* < 0.001) in red blood cell (RBC) and platelet (Plt) levels, while the latter (NAT (250 mg/kg)) showed a significant change (*p* < 0.01) in WBC and hemoglobin levels. When compared to arthritic control rats, those given NAT (250 mg/kg) showed statistically significant changes (*p* < 0.05) in RBC and Hb levels and significant decreases (*p* < 0.05) in WBC and platelets. However, the hematological changes induced by FCA were lessened more by indomethacin (10 mg/kg) than by NAT (250 mg/kg and 500 mg/kg) treatment, as shown in Figure 4.

#### 3.3.9 Effect of NAT on TNF-α, IL-10, and COX-2

**FIGURE 5:**
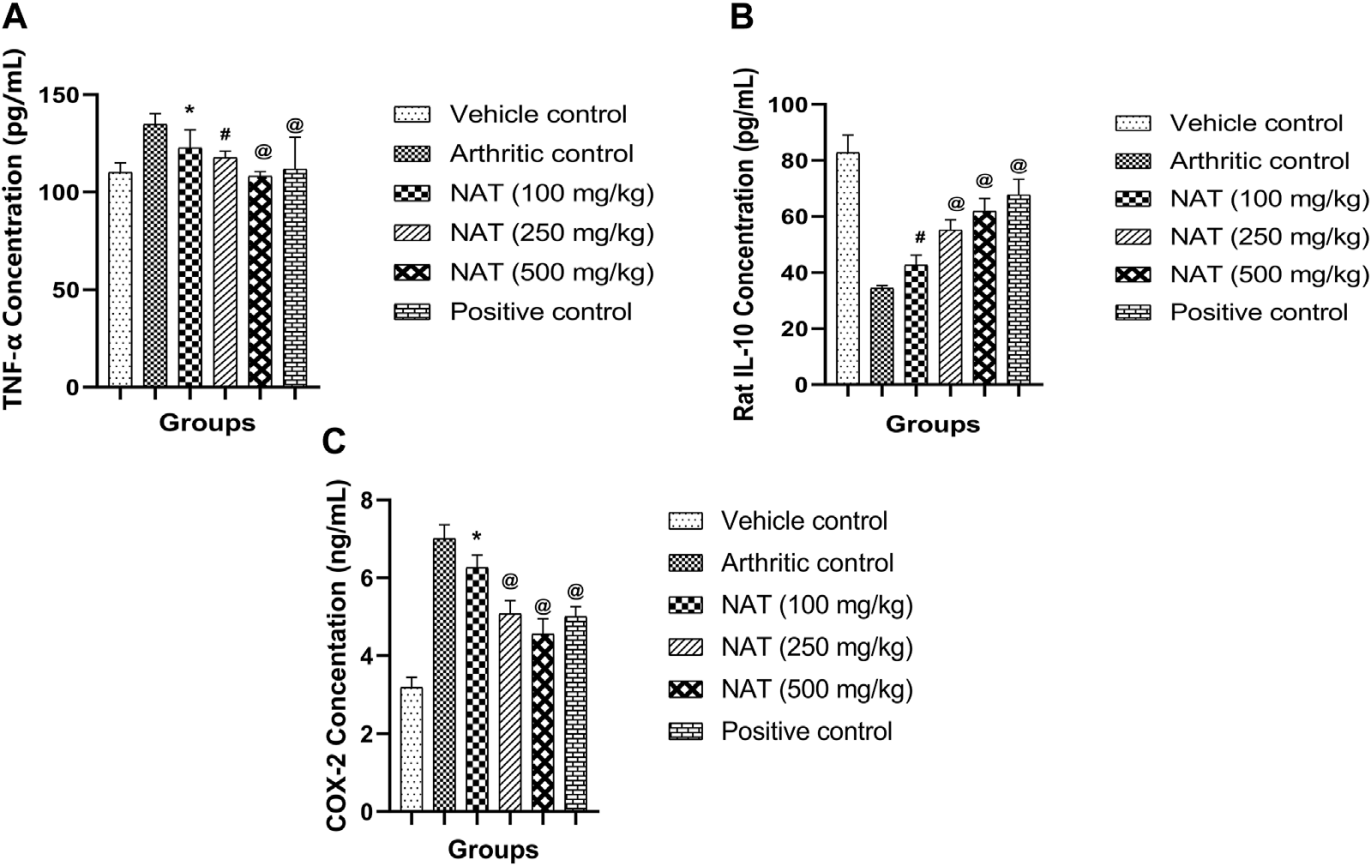
Effect of *Nyctanthes arbor-tristis* extract on cytokines: TNF-α **(A)**, IL-10 **(B)**, COX-2 **(C)** production in serum. Values are expressed as mean ± SEM for six animals. ∗ = < 0.05, # = < 0.01, @ = < 0.001 when compared to arthritic control. We performed a one-way analysis of variance followed by the Dunnett test.

TNF- α and COX-2, two pro-inflammatory cytokines, and IL-10, an anti-inflammatory cytokine, are crucial in the pathogenesis of RA. To determine the levels of TNF- α, IL-10, and COX-2 cytokines in the serum of arthritic rats, an analysis was conducted. The results are depicted in Figure 5. Rats with FCA-induced arthritis had significantly higher levels of TNF- α, IL-10, and COX-2 (*p* < 0.01). However, the elevated serum levels of TNF- α, IL-10, and COX-2 were decreased in the arthritic rats treated with NAT. This impact was nearly identical to that of regular indomethacin. While serum TNF- α, IL-10, and COX-2 levels were reduced in arthritic rats treated with NAT compared to the controls, their level was still higher than that of both NAT and the standards (*p* < 0.05).

#### 3.3.10 Histopathological study of NAT-treated rats

**FIGURE 6:**
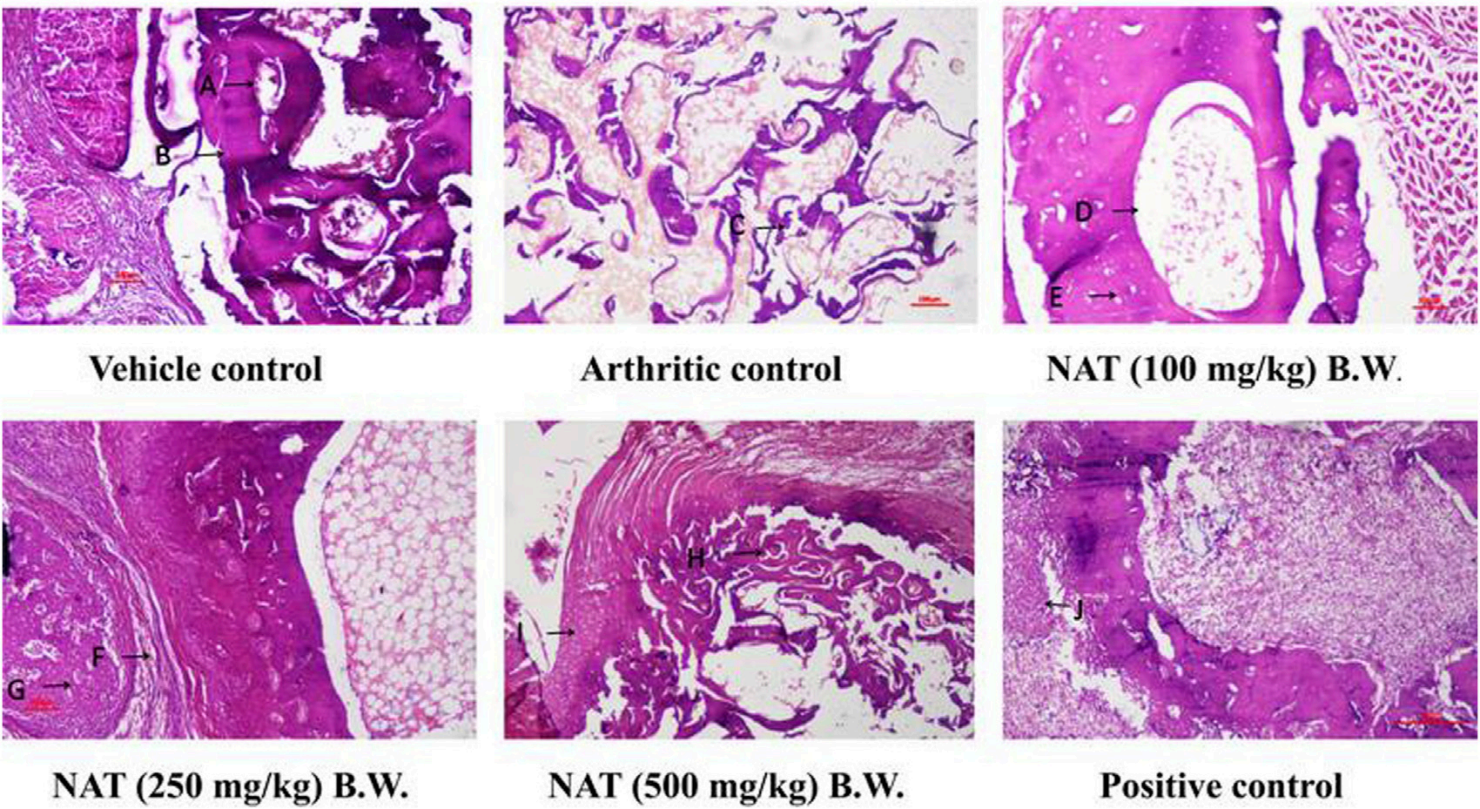
Evaluation of the extract of *Nyctanthes arbor-tristis* on the ankle joint of arthritic rats injected with FCA. Vehicle control: adequate no. of osteocytes in bone matrix **(A)** osteoblasts surrounding the border of the matrix **(B)**. H&E x 100x, arthritic control: disintegrated bone matrix with vacant lacuna containing no osteocyte **(C)**.H&E x 100x, NAT (100 mg/kg) BW: very few osteocyte lacunae in bone marrow matrix **(D)** surrounding the yellow bone marrow **(E)**. H&E x 100, NAT (250 mg/kg) BW: decreased bone marrow surrounded with fibrous tissue **(F)**. Osteocytes present in deep trabeculae. The proliferation of osteoblastic cells lining the border of the bone matrix **(G)**. H&E x 100 NAT (500 mg/kg) BW: decreased size and no. of trabeculae bone matrix **(H)**. Hyperplastic chondrocyte with increased activity **(I)**. H&E x 100, positive control: hyperplastic chondrocyte with increased activity **(J)** H&E x 100.

Rats with normal ankle joints showed intact articular cartilage, normal synovial tissue, and joint space free of inflammation upon histopathological examination. Additionally, NAT (250 mg/kg) and NAT (500 mg/kg) administration significantly reduced the arthritic control animals’ ankle joint modifications, such as prominent synovial lining hyperplasia, noticeable synoviocyte proliferation, inflammatory cell infiltration into joint capacity, pannus invasion of subchondral bone with subsequent articular cartilage, and bone erosion. Additionally, NAT (100 mg/kg) treatment slightly decreased the histopathological signs of arthritis. Furthermore, rats treated with NAT (500 mg/kg) showed significant histopathological changes. The information also demonstrates that oral administration of indomethacin to control rats with arthritis significantly protected them from histopathological changes (Figure 6).

## 4 Discussion

*Nyctnathes arbor-tristis* has been suggested as a potential medicinal agent for the treatment and prevention of painful joints and rheumatism. In our earlier studies, we found a significant activity of NAT hydroethanolic extract in *in vitro and* in silico studies (Sharma et al., 2023). The current study demonstrates that hydroethanolic extract of the aerial part of *Nyctnathes arbor-tristis* protects against adjuvant-induced arthritis in rats, an extensively used inflammatory arthritic animal model that replicates the pathological and clinical features of human RA. Following an injection of FCA, the inflammatory reaction typically starts between 1 and 3 days in the form of initial lesions, and then secondary lesions appear between 11 and 14 days later. Edema has been linked to increased vascular permeability, extravasation of fluid and proteins, and cellular invasion at the inflammatory sites (Bose et al., 2014). According to our analysis, the disease control group showed signs of persistent inflammation up to day 28 due to persistent edema and cellular invasion. In contrast to arthritic control rats, however, the treated animals showed remarkable protection against the morphological abnormalities of RA, with peak inflammation occurring up to the third day, followed by a rapid decline in paw edema. This decrease in edema may be related to a reduction in the production of prostaglandins as well as an increase in the invasion of neutrophils. Another study (Qasim et al., 2020) reported that the reduction in prostaglandin production corresponds to an elevation in neutrophil infiltration. The animal groups subjected to NAT treatments exhibited a notable increase in rat body weight when compared to the arthritic control group. The decrease in nutrient absorption from the intestine in FCA-challenged rats resulted in a significant reduction in rat body weight (Allawadhi et al., 2022). Similar to the effects observed with standard medications such as indomethacin, NAT substantially restored the rats’ body weight, possibly due to an improvement in their arthritic condition, which could subsequently normalize nutrient absorption from the intestine. These results align well with previous findings on the treatment of Freund’s complete adjuvant-induced arthritis using a cerium oxide nanoparticle (Akhtar et al., 2022).

In cases of adjuvant arthritis, the spleen plays a crucial role in generating cells and antibodies responsible for immunological responses. The spleens of arthritic rats exhibit an increase in cellularity (Khanfar et al., 2022). There is an observed rise in spleen cell weight in arthritic rats in the present study. We observed a clear increase in the organ weights of the spleen and thymus in FCA-induced rats. The administration of NAT led to a decrease in spleen and thymus weights, likely due to the suppression of splenic lymphocytes and the inhibition of lymphocyte infiltration into the synovium. These findings are consistent with those of earlier research involving the treatment of FCA-induced arthritic rats using β-sitosterol-loaded solid lipid nanoparticles (Zhang et al., 2020).

Further confirmation of the anti-arthritic capabilities of NAT and indomethacin was achieved through the evaluation of several biochemical markers in rat serum. These markers, including AST, ALT, CRP, and RF, serve as crucial indicators for assessing the anti-arthritic potential of the drug. Reduced RF and CRP levels, with RF serving as a potential marker for RA, were characterized by a notable increase in distal interphalangeal arthritis incidence (Sharma et al., 2023). Additionally, a persistently high serum CRP level is recognized as a strong indicator of RA (Petchi et al., 2015). Elevated serum levels of AST, ALT, and ALP were observed, while the total protein level decreased. Evaluating serum AST, ALT, and ALP levels offers a straightforward and effective method for assessing the drug’s anti-arthritic activity. Aminotransferase and ALP activities notably increased in arthritic rats, serving as reliable indicators of liver and kidney impairment, which is a known feature of adjuvant arthritis. It is worth noting that serum AST and ALT have been reported to play a significant role in the generation of biologically active chemical mediators, such as bradykinins, in the inflammatory process (Kadam and Bodhankar, 2013). In this investigation, the treatment of arthritic rats with NAT and indomethacin resulted in a significant reduction in the elevated levels of AST, ALT, CRP, and RF as compared to arthritic control rats. These findings suggest the therapeutic potential of the studied NAT in RA treatment. Additionally, the significant decrease in the elevated serum levels of AST and ALT indicates that the studied fractions did not induce liver injury in the rats. In summary, the results of lysosomal enzyme measurements are consistent with prior reports in the literature regarding *Calotropis procera* leaves (Singh et al., 2021).

Anemia is a prevalent condition in rheumatoid arthritis, characterized by reduced levels of red blood cells (RBCs) and hemoglobin (Hb) content (Mahnashi et al., 2021). This anemic state arises from abnormalities in iron storage within the synovial tissue and the reticuloendothelial system (Das et al., 2021). Furthermore, the reduced capacity of the bone marrow to produce sufficient blood cells is another contributing factor to the decline in RBC and Hb levels (Hess and D’Alessandro, 2022). In RA, immune system activation in response to antigenic assaults results in elevated white blood cell (WBC) and platelet levels (Manan et al., 2020). An increase in WBC count is linked to heightened immune system activity against destructive pathogens, ultimately triggering the activation of inflammatory signaling molecules (Ge et al., 2021). Platelets play a significant role in inflammation and immunomodulation, as microparticles released from platelets interact with WBCs and contribute to systemic and joint inflammation in RA (Harifi and Sibilia, 2016). Treatment with NAT led to an increase in RBC and Hb levels and reduced WBC and platelet levels when compared to the arthritic control group, thereby confirming the anti-arthritic potential of NAT. The outcomes of the hematological assessments closely mirrored the findings previously documented in the literature for the extract derived from methanolic, n-hexane, and ethyl acetate fractions of the bark of *Acacia modesta* (Mashaal et al., 2023).

It was hypothesized that administering FCA stimulates T cells, which in turn excite macrophages and monocytes, leading to an upregulation of lysosomal enzymes and the release of pro-inflammatory cytokines. Arthritic joints are characterized by the uncontrolled spread of synovial tissue, joint dysfunction, tissue destruction, bone erosion, and programmed cell death, all of which may be traced back to the overexpression of pro-inflammatory cytokines (Komatsu and Takayanagi, 2012). In this study, we used ELISA to examine the impact of NAT hydroethanolic extract on blood COX-2 and TNF-α levels. In this research, rats treated with indomethacin and NAT showed a significant decrease in elevated TNF-α levels seen in disease control rats, indicating the potential of the plant to reduce arthritis and inflammation. The transcription factor NF-кB was shown to regulate TNF-α expression, whereas TNF-α itself was shown to act as a potent stimulator of NF-кB (Makrov, 2001). Because NF-кB is required to produce pro-inflammatory cytokines, reducing TNF-α has a net beneficial impact of reducing their levels (Wong et al., 2008). Current research has shown that the plant extract significantly suppressed TNF-α expression, which may have resulted from a decrease in NF-кB expression levels, as was previously observed. Additionally, in rheumatoid synovium, the COX-2 (cyclooxygenase) pathway’s generation of PGE2 through arachidonic acid metabolism is particularly important (Cheng et al., 2015). Cartilage and bone erosions, fluid extravasation, discomfort, vasodilation, and similar conditions may all be accelerated by elevated PGE2 concentrations (Fattahi and Mirshafiey, 2012). While the current investigation found elevated COX-2 levels in arthritic control animals, a substantial decrease was seen in NAT-administered rats, suggesting that NAT may have protected inflamed joints from further damage by reducing prostaglandin production as a result of subdual COX-2 generation. So NAT’s anti-arthritic impact may also be due to its potential to block the metabolism of arachidonic acid. As an immunomodulatory cytokine, IL-10 alters the progression of RA synovitis (Sharma et al., 2023). In RA pathogenesis, IL-10 does more than only inhibit the production of Th1 cell-generated cytokines (GM-CSF, IL-1, IFN-γ, and TNF-α); it preserves joint tissue, prevents the activity of antigen-presenting cells, and suppresses the production of IL-18 mRNA (Lin et al., 2013). Arthritic control rats showed a large decrease in IL-10 levels, whereas all treatment groups, including those given indomethacin, showed a significant increase, suggesting an anti-inflammatory/immunomodulatory effect of NAT in this condition. These findings align with the outcomes documented for the *Ephedra gerardiana* aqueous ethanolic extract and fractions, as reported in a previous study (Uttra et al., 2019).

Radiographic images are employed for the assessment of tissue swelling, erosions, and joint deformities in arthritis patients. These images provide insight into soft tissue lesions, often serving as early indicators of arthritis. Additionally, bone erosion and the deterioration of trabecular bone are characteristic pathological changes observed in human arthritis (Almarestani et al., 2011). In the case of FCA-induced arthritic rats, there was a noticeable presence of soft tissue swelling and a narrowing of joint spaces, indicating bone damage associated with arthritic conditions. The radiographic observations of the treatment groups receiving NAT demonstrated mitigation of arthritis-related joint alterations are given in Figure 1.

The histopathological findings also indicated that NAT possesses the ability not just to reduce inflammation but also to shield cartilage and bones from erosion. These observations complement the data acquired from other biochemical measurements. Arthritis is characterized by the infiltration of inflammatory cells, subcutaneous inflammation, and synovial inflammation (Vishnu and Krishnan, 2018). These findings were consistent with the outcomes reported in studies involving the treatment of rats with complete Freund’s adjuvant-induced arthritis using chrysin (Faheem et al., 2023).

## 5 Conclusion

According to the findings, oral administration of *Nyctanthes arbor-tristis* to arthritic rats significantly reduced paw edema and restored body weight; re-established an altered biochemical and hematological profile and inflammatory mediators’ serum expression levels; and decreased bone and cartilage destruction. Hence, the capacity of the tested plant to reduce TNF-α, increase IL-10 levels, and reduce the concentrations of the inflammatory enzyme COX-2 seems to be linked to its anti-rheumatic and immunomodulatory capabilities. As a result, *Nyctanthes arbor-tristis* may be the best strategy to treat RA. However, further research is essential to pinpoint and separate the potential phytoconstituents responsible for the anti-arthritic potential, thus enabling the use of *Nyctanthes arbor-tristis* in the treatment of arthritic conditions.

## Data Availability

The original contributions presented in the study are included in the article/Supplementary material; further inquiries can be directed to the corresponding authors.
